# Boswellic Acid Enhances Gemcitabine’s Inhibition of Hypoxia-Driven Angiogenesis in Human Endometrial Cancer

**DOI:** 10.3390/medicina61122181

**Published:** 2025-12-08

**Authors:** Senem Alkan Akalın, Yasemin Afşin, İlhan Özdemir, Mehmet Cudi Tuncer, Şamil Öztürk

**Affiliations:** 1 Division of Gynecology and Obstetrics, Private Medical Practice, Bursa 16990, Türkiye; drsakalin@hotmail.com; 2 Gynecology and Obstetrics, Private Batman Life Hospital, Batman 72040, Türkiye; dryaseminafsin@outlook.com; 3 Department of Histology Embryology, Faculty of Medicine, Kahramanmaraş Sütçü İmam University, Kahramanmaraş 46100, Türkiye; ilhanozdemir25@yandex.com; 4 Department of Anatomy, Faculty of Medicine, Dicle University, Diyarbakır 21280, Türkiye; 5 Vocational School of Health Services, Çanakkale Onsekiz Mart University, Çanakkale 17100, Türkiye; ozturksamil@outlook.com

**Keywords:** endometrial cancer, boswellic acid, gemcitabine, hypoxia, angiogenesis, apoptosis, HIF-1α, VEGF, Caspase-3

## Abstract

*Background and Objectives*: Endometrial carcinoma is among the most common gynecological malignancies, with recurrence and chemoresistance remaining major clinical challenges. This study aimed to evaluate the combined effects of Boswellic acid (BA), a natural pentacyclic triterpene, and Gemcitabine (GEM), a nucleoside analog chemotherapeutic, on hypoxia, angiogenesis, and apoptosis in human endometrial cancer cells. *Materials and Methods*: ECC-1 cells were treated with BA, GEM, or their combination under normoxic and hypoxic conditions. Cell viability (MTT assay); nuclear morphology (NucBlue staining); cell cycle distribution (PI flow cytometry); angiogenesis (VEGF ELISA expression); apoptosis (Caspase-3/7 activity; Bax; Bcl-2 expression); inflammatory cytokines (IL-1β; IL-6; TNF-α); and gene ontology enrichment were analyzed. *Results*: Both BA and GEM reduced cell viability in a dose- and time-dependent manner, with the combination producing synergistic cytotoxicity and lower IC_50_ values. Hypoxia enhanced drug sensitivity, particularly in combination therapy. BA and GEM significantly suppressed HIF-1α and VEGF expression, with maximal inhibition observed in the combination group. Apoptotic induction was confirmed by increased Bax and Caspase-3 and decreased Bcl-2 expression, together with elevated Caspase-3/7, -8, and -9 activity. Pro-inflammatory cytokine levels were markedly reduced, and gene ontology analysis revealed enrichment of apoptotic, anti-proliferative, and anti-angiogenic pathways. *Conclusions*: BA + GEM combination synergistically suppresses hypoxia-driven angiogenesis and promotes apoptosis in endometrial cancer cells. These findings support its potential as an adjuvant therapeutic approach, warranting further preclinical and clinical validation.

## 1. Introduction

Endometrial cancer is one of the most common gynecological malignancies in women worldwide, and its incidence continues to rise, particularly in developed countries [1]. Although early-stage disease generally has a favorable prognosis, recurrence and resistance to chemotherapy in advanced stages present major clinical challenges, significantly limiting long-term survival [2]. This underscores the urgent need for novel therapeutic approaches that can overcome treatment resistance and improve outcomes.

GEM, a nucleoside analog that interferes with DNA synthesis, is widely used as an effective chemotherapeutic agent in the treatment of solid tumors [3]. However, its clinical efficacy is often limited due to intrinsic and acquired resistance, as well as dose-dependent toxicities, which restrict its use as monotherapy [4]. As a result, combination therapies are preferred to improve treatment response. In this context, the integration of natural compounds with conventional chemotherapeutics has emerged as a promising strategy to suppress tumor progression while potentially reducing side effects [5].

BA, a pentacyclic triterpene derived from *Boswellia serrata* resin, has demonstrated anti-inflammatory, antioxidant, and anticancer properties [6]. Preclinical evidence indicates that BA sensitizes gastric cancer cells to cisplatin-induced apoptosis via p53 signaling [7] and modulates angiogenesis and survival pathways in other malignancies [8]. These multimodal activities highlight its potential as an adjuvant compound in cancer therapy.

Hypoxia and angiogenesis are recognized as critical hallmarks of endometrial cancer progression. Hypoxia-inducible factor-1α (HIF-1α) is a key regulator of cellular adaptation to low-oxygen conditions, while vascular endothelial growth factor (VEGF) and its receptor VEGFR2 drive tumor vascularization and correlate with poor prognosis [9]. Hypoxia is highly prevalent in endometrial carcinomas and represents a critical driver of tumor progression, angiogenesis, and therapeutic resistance. Clinical and histopathological analyses have demonstrated that hypoxic endometrial tumors with increased vascular density are significantly associated with poorer disease-specific and distant disease-free survival, emphasizing the prognostic impact of hypoxia-induced angiogenesis [10]. Furthermore, recent findings indicate that a higher proportion of HIF-1α–positive immune cells correlates with higher histologic grade and diffuse GLUT-1 expression patterns in endometrial cancer, suggesting that hypoxia-regulated proteins contribute to tumor aggressiveness and may serve as potential prognostic biomarkers [11]. Collectively, these data highlight the biological and clinical importance of targeting hypoxia-driven molecular mechanisms in endometrial cancer. Targeting these pathways is therefore considered a rational approach to enhance therapeutic efficacy.

Based on these considerations, the present study investigated the effects of BA and GEM, individually and in combination, on key molecular pathways driving endometrial cancer progression. Unlike our previous investigations involving flavonoid-based compounds such as hesperidin in combination with chemotherapeutics, this study introduces BA (a pentacyclic triterpenoid) as a structurally and mechanistically distinct natural agent. Furthermore, the experiments were performed under hypoxic conditions (1% O_2_) to closely mimic the tumor microenvironment and to explore the modulation of HIF-1α/VEGF signaling, which was not evaluated in our earlier normoxic studies.

The use of ECC-1 endometrial carcinoma cells, representing a different molecular subtype from models such as ISHIKAWA, also provides additional insight into tumor heterogeneity and response variability. Collectively, this study provides the first demonstration that BA synergizes with GEM to suppress hypoxia-driven angiogenesis and promote apoptosis in endometrial cancer cells, highlighting its novel contribution beyond previously reported flavonoid-based synergistic models.

## 2. Material and Methods

The assays selected in this study were chosen to comprehensively evaluate the effects of BA and GEM on key molecular pathways implicated in hypoxia-driven angiogenesis and apoptosis. MTT and cell-cycle analyses were used to assess proliferation and cytotoxicity; VEGF ELISA and HIF-1α/HIF-dependent markers were used to examine hypoxia-induced angiogenesis; caspase activity, Bax/Bcl-2, and Annexin V assays assessed apoptosis activation through intrinsic and extrinsic pathways; cytokine profiling evaluated inflammatory modulation; and GO enrichment analysis was performed to identify integrated biological pathways modulated by BA and GEM.

### 2.1. Cell Culture and Maintenance of ECC-1 Endometrial Cancer Cells

The human endometrial adenocarcinoma cell line (ECC-1; CRL-2923, ATCC, Manassas, VA, USA) was cultured under standard conditions. Cells were maintained in Dulbecco’s Modified Eagle Medium (DMEM; Gibco, Thermo Fisher Scientific, Waltham, MA, USA) supplemented with 10% fetal bovine serum (FBS; Gibco, Thermo Fisher Scientific, Waltham, MA, USA) and 1% penicillin–streptomycin (100 U/mL penicillin and 100 µg/mL streptomycin; Gibco, Thermo Fisher Scientific, Waltham, MA, USA). Cultures were incubated at 37 °C in a humidified atmosphere containing 5% CO_2_.

The ECC-1 human endometrial adenocarcinoma cell line (CRL-2923, ATCC, Manassas, VA, USA) was authenticated by short tandem repeat (STR) profiling prior to experiments and routinely tested for mycoplasma contamination using the MycoAlert™ Mycoplasma Detection Kit (Lonza, Basel, Switzerland). Cells were used within passages 3–10 to ensure genomic stability.

### 2.2. Preparation and Application of BA and GEM

BA (BA; 3-O-acetyl-11-keto-β-boswellic acid, Sigma-Aldrich, St. Louis, MO, USA) was dissolved in dimethyl sulfoxide (DMSO; Sigma-Aldrich, St. Louis, MO, USA) to prepare a stock solution and further diluted with culture medium to the desired concentrations. The final concentration of DMSO in culture medium did not exceed 0.1%. GEM (GEM; Sigma-Aldrich, St. Louis, MO, USA) was dissolved in sterile distilled water prior to use. Cells were divided into single-agent treatment groups (BA or GEM) and a combination group (BA + GEM) and were incubated for 24 h and 48 h. Control cells received only the corresponding solvent.

### 2.3. Induction of Hypoxia Using a Controlled Gas Incubator

To mimic hypoxic conditions, ECC-1 cells were incubated in a tri-gas incubator (Galaxy_®_ 48R, Eppendorf, Hamburg, Germany) supplied with a premixed gas cylinder (1% O_2_, 5% CO_2_, and 94% N_2_; Linde Gas, Munich, Germany) for 24 h. Normoxic controls were maintained at 21% O_2_ in a standard humidified incubator (Thermo Fisher Scientific, Waltham, MA, USA). The successful induction of hypoxia was functionally verified by the upregulation of HIF-1α and VEGF mRNA expression, as determined by qRT-PCR analysis, both of which are established transcriptional markers of cellular hypoxic response.

### 2.4. MTT Cell Viability Assay

The cytotoxic effects of the treatments were evaluated using the 3-(4,5-dimethylthiazol-2-yl)-2,5-diphenyltetrazolium bromide (MTT) assay (Sigma-Aldrich, St. Louis, MO, USA). ECC-1 cells were seeded into 96-well plates (Corning Inc., Corning, NY, USA) and treated with the indicated experimental conditions. Following treatment, MTT reagent (5 mg/mL in PBS) was added to each well and incubated for 4 h at 37 °C. The resulting formazan crystals were solubilized in dimethyl sulfoxide (DMSO; Sigma-Aldrich, St. Louis, MO, USA), and absorbance was measured at 570 nm using a Multiskan GO microplate spectrophotometer (Thermo Fisher Scientific, Waltham, MA, USA). All experiments were performed in triplicate (n = 3).

Dose–response curves were generated from cell viability data, and half-maximal inhibitory concentration (IC_50_) values were calculated using nonlinear regression (log(inhibitor) vs. response, variable slope) in GraphPad Prism version 9.0 (GraphPad Software, San Diego, CA, USA). Each IC_50_ value was derived from at least three independent experiments performed in triplicate.

Drug interaction effects between BA and GEM were evaluated using the Chou–Talalay method. Combination index (CI) values were calculated based on the median-effect equation with CompuSyn software version 1.0 (ComboSyn Inc., Paramus, NJ, USA). CI < 1.0 indicated synergism, CI = 1.0 indicated additivity, and CI > 1.0 indicated antagonism. Isobologram plots were generated to visualize the nature of the drug interactions.

### 2.5. Nuclear Morphology Analysis Using Phase-Contrast and NucBlue (DAPI) Staining

NucBlue™ Live ReadyProbes™ Reagent (Hoechst 33342; Invitrogen, Thermo Fisher Scientific, Eugene, OR, USA) was used to assess nuclear morphology. ECC-1 cells were cultured in 6-well plates (Corning Inc., Corning, NY, USA) and treated according to the designated experimental groups, followed by gentle washing with phosphate-buffered saline (PBS; Gibco, Thermo Fisher Scientific, Waltham, MA, USA). NucBlue solution (1 drop per 1 mL of culture medium), prepared according to the manufacturer’s instructions, was added to each well, and the cells were incubated for 20 min at 37 °C. After staining, cells were washed with PBS and examined under a fluorescence microscope equipped with a DAPI filter set (excitation/emission: 350/461 nm; Nikon Eclipse Ti2, Nikon Instruments, Tokyo, Japan). Apoptotic nuclear changes, such as chromatin condensation and fragmentation, were recorded, and representative images were captured using a digital camera system (DS-Qi2, Nikon Instruments, Tokyo, Japan).

### 2.6. Cell Cycle Distribution Analysis by Propidium Iodide (PI) Staining and Flow Cytometry

Cell cycle distribution was assessed using propidium iodide (PI) staining. Cells were fixed in 70% ethanol (Merck, Darmstadt, Germany) at −20 °C for 24 h and subsequently incubated with RNase A (100 µg/mL; Sigma-Aldrich, St. Louis, MO, USA) and PI (50 µg/mL; Sigma-Aldrich, St. Louis, MO, USA) for 30 min at room temperature in the dark. Samples containing at least 10,000 cells were analyzed using a BD FACSCanto™ II flow cytometer (BD Biosciences, San Jose, CA, USA). Cell cycle distribution (G0/G1, S, and G2/M phases) was determined with FlowJo software, version 10 (BD Biosciences, Ashland, OR, USA). All experiments were performed in triplicate (n = 3).

### 2.7. Quantification of Apoptosis by Annexin V-FITC and Propidium Iodide (PI) Double Staining Using Flow Cytometry

Apoptotic cell death was further evaluated using an Annexin V-FITC/Propidium Iodide (PI) Apoptosis Detection Kit (BD Biosciences, San Jose, CA, USA), following the manufacturer’s instructions. ECC-1 cells were seeded in 6-well plates (Corning Inc., Corning, NY, USA) and treated with BA, GEM, or their combination at IC_50_ concentrations for 48 h. After treatment, cells were harvested, washed twice with cold phosphate-buffered saline (PBS; Gibco, Thermo Fisher Scientific, Waltham, MA, USA), and resuspended in binding buffer. Annexin V-FITC and PI were added, and the samples were incubated for 15 min at room temperature in the dark. Fluorescence signals were analyzed with a BD FACSCanto™ II flow cytometer (BD Biosciences, San Jose, CA, USA), and data were processed using FlowJo software version 10 (BD Biosciences, Ashland, OR, USA). Cells were classified as viable (Annexin V^−^/PI^−^), early apoptotic (Annexin V^+^/PI^−^), late apoptotic (Annexin V^+^/PI^+^), or necrotic (Annexin V^−^/PI^+^). All experiments were performed in triplicate (n = 3).

### 2.8. Evaluation of Angiogenesis Through VEGF Quantification by ELISA

The angiogenic potential was assessed by quantifying VEGF expression using an enzyme-linked immunosorbent assay (ELISA). Cell culture supernatants were collected after drug treatment, and VEGF concentrations were measured with a Human VEGF Quantikine ELISA Kit (R&D Systems, Minneapolis, MN, USA; Cat. No. DVE00), according to the manufacturer’s instructions. Absorbance was recorded at 450 nm with wavelength correction at 570 nm using a Multiskan GO microplate reader (Thermo Fisher Scientific, Waltham, MA, USA). All experiments were performed in triplicate (n = 3).

Pro-inflammatory cytokines (IL-1β, IL-6, and TNF-α) were quantified due to their known roles in promoting hypoxia, angiogenesis, and tumor progression.

### 2.9. Caspase-3/7, -8, and -9 Activity Assay (ELISA Method)

ECC-1 cells were treated with BA, GEM, and the BA + GEM combination for 48 h. After treatment, cells were lysed using RIPA buffer containing protease inhibitors, and the supernatant was collected by centrifugation at 12,000× *g* for 10 min at 4 °C. Invitrogen Human Caspase-3/7, -8, and -9 ELISA Kits (Thermo Fisher Scientific, 168 Third Avenue, Waltham, MA, USA) were used to measure caspase activities according to the manufacturer’s instructions. One hundred microliters of each sample was added per well, and the colorimetric reaction was developed for 10 min at room temperature before adding the stop solution. Absorbance was measured at 450 nm using a Multiskan GO ELISA reader (Thermo Scientific, Waltham, MA, USA). Each sample was assayed in triplicate (n = 3) for statistical analysis.

### 2.10. Quantitative Real-Time PCR (qRT-PCR) Analysis of Hypoxia-, Angiogenesis-, and Apoptosis-Related Genes

The mRNA expression levels of HIF-1α, VEGF, Bax, Bcl-2, and Caspase-3 were determined using qRT-PCR. Total RNA was extracted with TRIzol reagent (Invitrogen, Thermo Fisher Scientific, Carlsbad, CA, USA) according to the manufacturer’s protocol. Complementary DNA (cDNA) was synthesized from 1 µg of total RNA using the High-Capacity cDNA Reverse Transcription Kit (Applied Biosystems, Foster City, CA, USA). qRT-PCR was carried out using PowerUp™ SYBR™ Green Master Mix (Applied Biosystems, Foster City, CA, USA) on a StepOnePlus™ Real-Time PCR System (Applied Biosystems, Foster City, CA, USA).

Gene-specific primers for HIF-1α, VEGF, Bax, Bcl-2, and Caspase-3 were designed using Primer-BLAST (NCBI) and synthesized by Sentegen Biotech (Ankara, Türkiye). GAPDH was used as the endogenous control. Relative gene expression was calculated using the 2^−ΔΔCt^ method. All experiments were performed in triplicate (n = 3).

The primer sequences used for amplification are listed in Table 1.

Gene Ontology enrichment analysis was performed using the Database for Annotation, Visualization and Integrated Discovery (DAVID) v2024Q1. Enriched GO terms were visualized and plotted using GraphPad Prism version 10 (GraphPad Software, San Diego, CA, USA). GO enrichment analysis was included to contextualize gene-level changes within broader biological pathways relevant to hypoxia, angiogenesis, and apoptosis.

### 2.11. Statistical Analysis of Experimental Data

All experiments were performed in at least three independent biological replicates (n ≥ 3). Each biological replicate contained three technical replicate measurements, and therefore all reported “n = 3” values refer to biological replicates unless otherwise specified. Data are expressed as the mean ± standard deviation (SD). Statistical analyses were conducted using GraphPad Prism version 10 (GraphPad Software, San Diego, CA, USA). Data distribution was first assessed for normality using the Shapiro–Wilk test. Differences between multiple groups were evaluated by one-way analysis of variance (ANOVA), followed by Tukey’s post hoc test for pairwise comparisons. A *p*-value of <0.05 was considered statistically significant.

Gene Ontology (GO) enrichment analysis was performed using the Database for Annotation, Visualization and Integrated Discovery (DAVID) v2024Q1. Terms with adjusted *p* < 0.05, false discovery rate (FDR) < 0.25, and fold change > 2 were considered statistically significant. Enriched GO terms were visualized and plotted using GraphPad Prism version 10 (GraphPad Software, San Diego, CA, USA). The complete list of significant GO terms is provided in Supplementary Files S1 and S2.

## 3. Results

### 3.1. Cytotoxic Effects of BA and GEM Assessed by MTT Assay

BA and GEM significantly reduced the viability of endometrial cancer cells in a dose- and time-dependent manner. Combination treatment with BA + GEM produced markedly greater cytotoxicity compared with either agent alone (*p* < 0.01).

For BA, a significant reduction in cell viability was observed at concentrations of 25, 50, and 100 µM after 24 h of treatment compared with the control group (*p* < 0.001), whereas 10 µM showed no significant effect (*p* > 0.05). After 48 h, BA decreased viability significantly at all tested concentrations starting from 10 µM (*p* < 0.001). The calculated IC_50_ values were 41.6 µM at 24 h and 34.7 µM at 48 h.

GEM demonstrated stronger cytotoxicity than BA, with significant reductions in viability at all concentrations ≥ 1 µM after both 24 h and 48 h of exposure (*p* < 0.001). The IC_50_ values for GEM were 1.29 µM at 24 h and 1.04 µM at 48 h.

The BA + GEM combination showed the most potent effect, with rapid decreases in cell viability beginning at lower combination doses. At 24 h, viability fell below 10% at the highest dose, and at 48 h it dropped to 16%, remaining under 20% in all high-dose groups. The combination achieved lower IC_50_ values than either single agent, indicating a synergistic interaction.

**Figure 1:**
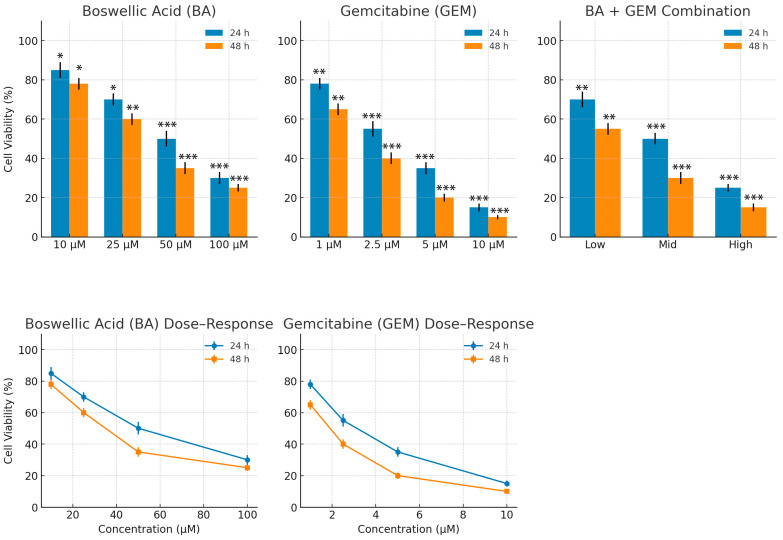
Effects of BA, GEM, and their combination on ECC-1 cell viability at 24 and 48 h. Cells were treated with increasing concentrations of BA (10–100 µM), GEM (1–10 µM), or BA + GEM combinations (Low, Mid, and High). The combination treatment significantly reduced cell viability in a dose- and time-dependent manner compared with single treatments. Data are expressed as mean ± SD (n = 3). Statistical significance was determined by one-way ANOVA followed by Tukey’s post hoc test (* *p* < 0.05, ** *p* < 0.01, *** *p* < 0.001).

**Figure 2:**
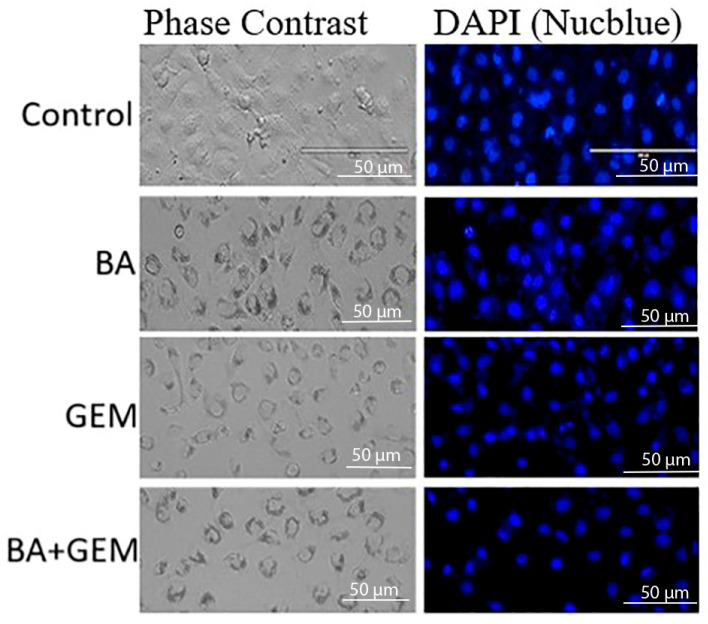
Nuclear morphology of ECC-1 cells following treatment with BA, GEM, and their combination (BA + GEM). Cells were exposed to BA (34.7 µM), GEM (1.04 µM), or BA + GEM (IC_50_ concentrations) for 48 h. Phase-contrast images (**left**) show cellular morphology, while NucBlue (DAPI) staining (**right**) reveals nuclear features. Control cells displayed intact, round nuclei with uniform staining. BA-treated cells exhibited moderate chromatin condensation and nuclear shrinkage, whereas GEM-treated cells showed more pronounced nuclear fragmentation. The BA + GEM combination induced the most extensive apoptotic changes, characterized by strong condensation and nuclear fragmentation. Images are representative of three independent experiments.

Collectively, these findings demonstrate that both BA and GEM reduce the viability of endometrial cancer cells in a time- and dose-dependent manner, with GEM exhibiting higher potency, while their combination exerts a synergistic cytotoxic effect (Figure 1 and Figure 2).

### 3.2. Effects of BA and GEM on Cell Viability Under Normoxic and Hypoxic Conditions

Under normoxic conditions, cell viability was highest in the control group (100%), while treatment with BA and GEM reduced viability to 85% and 70%, respectively. The BA + GEM combination further decreased viability to 55%, representing the most pronounced cytotoxic effect under normoxia.

Exposure to hypoxic conditions reduced viability across all groups. Control cells decreased to 80%, BA-treated cells to 60%, GEM-treated cells to 50%, and the BA + GEM combination to 30%. Thus, hypoxia enhanced the sensitivity of ECC-1 cells to both single-agent and combination treatments, with the combined regimen showing the greatest effect.

**Figure 3:**
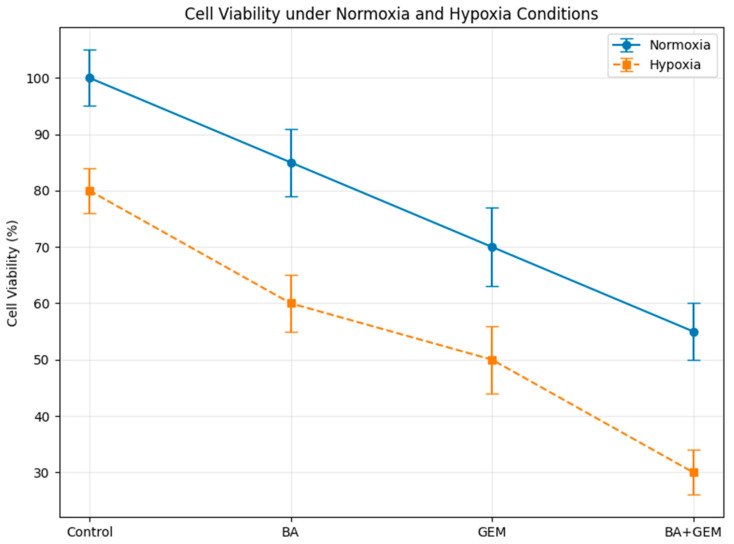
Effect of BA, GEM, and their combination on ECC-1 cell viability under normoxic and hypoxic conditions. Cells were treated with IC_50_ concentrations of BA (34.7 µM), GEM (1.04 µM), and BA + GEM for 48 h. The combination treatment significantly reduced viability under both normoxia and hypoxia, with the strongest inhibitory effect observed in hypoxic cells. Data are expressed as mean ± SD (n = 3). Statistical significance was determined by one-way ANOVA followed by Tukey’s post hoc test (*p* < 0.05).

A direct comparison of normoxia and hypoxia revealed that hypoxia significantly reduced absolute viability in all groups, although the relative order of treatment effects remained unchanged (Control > BA > GEM > BA + GEM). These findings indicate that hypoxia potentiates the anti-proliferative effects of BA and GEM in endometrial cancer cells (Figure 3).

### 3.3. Inhibition of Angiogenesis by BA and GEM: VEGF Expression Analysis

**Figure 4:**
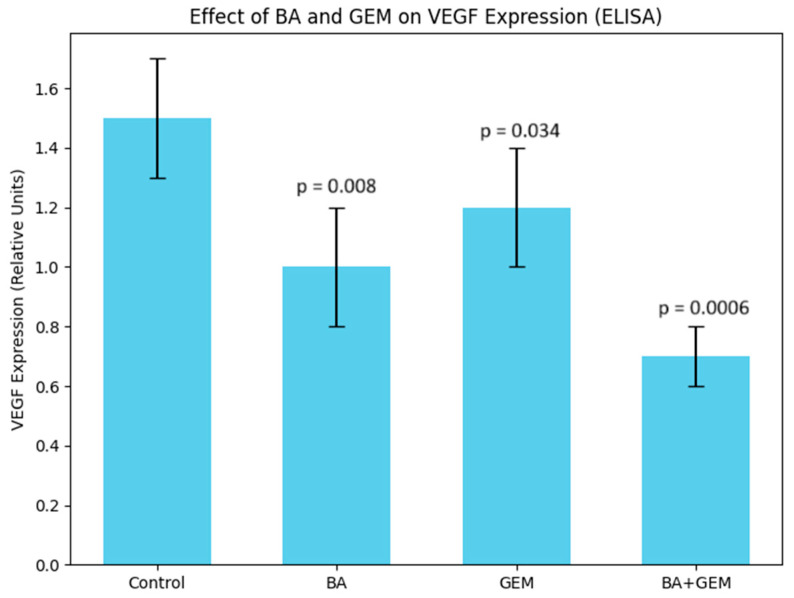
Effect of BA, GEM, and their combination (BA + GEM) on VEGF secretion in ECC-1 cells, assessed by ELISA. Control cells exhibited high VEGF expression (1.50 ± 0.15). BA significantly reduced VEGF secretion to 1.00 ± 0.10 (*p* = 0.008 vs. control), while GEM decreased VEGF to 1.25 ± 0.12 (*p* = 0.034 vs. control). The BA + GEM combination produced the strongest inhibition (0.70 ± 0.08; *p* = 0.0006 vs. control). Data represent mean ± SD from three independent experiments (n = 3). Statistical analysis was performed by one-way ANOVA followed by Tukey’s post hoc test.

Angiogenic potential was evaluated by measuring VEGF secretion using ELISA. Control cells exhibited high VEGF levels, consistent with the pro-angiogenic phenotype of untreated ECC-1 cells. Treatment with BA resulted in a significant reduction in VEGF expression, whereas GEM alone caused a moderate but measurable suppression. Importantly, the BA + GEM combination produced the most pronounced inhibitory effect, leading to a marked decrease in VEGF levels compared with either agent alone (*p* < 0.01). These findings indicate that BA and GEM synergistically inhibit angiogenesis by downregulating VEGF signaling, thereby contributing to their combined cytotoxic and anti-proliferative activity (Figure 4).

**Figure 5:**
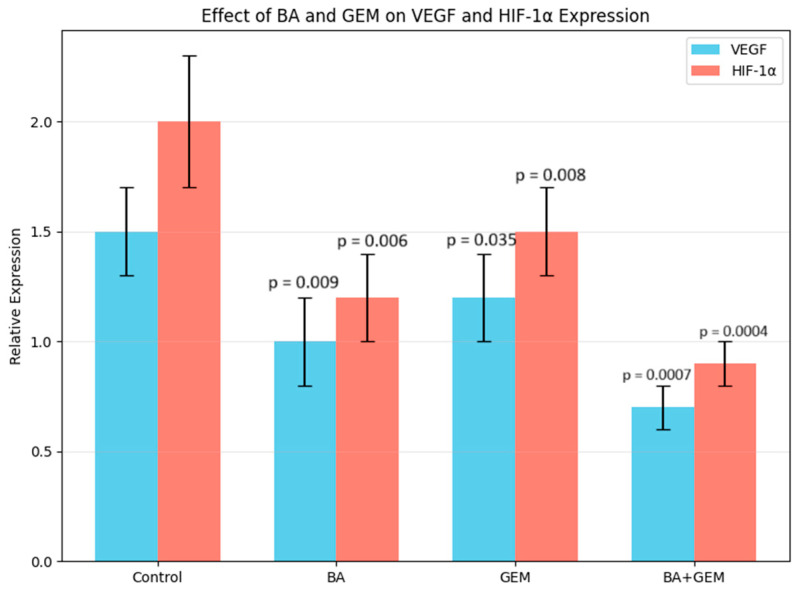
Relative mRNA expression levels of VEGF and HIF-1α in ECC-1 cells treated with BA, GEM, or their combination for 48 h, as measured by qRT-PCR. Both genes were significantly downregulated by all treatments, with the most pronounced suppression observed in the BA + GEM group. Data represent mean ± SD (n = 3). Statistical analysis was performed using Welch’s *t*-test (*p* < 0.05).

Molecules associated with angiogenesis were inhibited. VEGF and HIF-1α expression levels were highest in control cells but were significantly reduced by BA and GEM treatments. BA treatment decreased both markers at the *p* < 0.01 level (**), while GEM reduced VEGF at *p* < 0.05 (*) and HIF-1α at *p* < 0.01 (**). The BA + GEM combination induced the strongest suppression of VEGF and HIF-1α, with changes highly significant at *p* < 0.001 (**), indicating a synergistic inhibitory effect on angiogenesis-related molecules (Figure 5).

**Figure 6:**
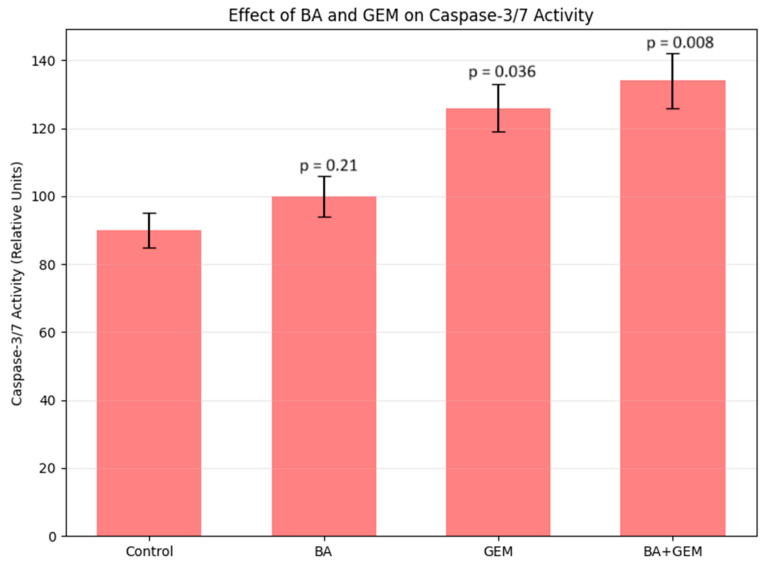
Effect of BA, GEM, and their combination on Caspase-3/7 activity in ECC-1 endometrial cancer cells after 48 h of treatment. Caspase-3/7 activity was measured using a colorimetric ELISA and expressed as absorbance at 450 nm. The BA + GEM combination markedly enhanced Caspase-3/7 activation compared with single treatments, confirming a synergistic induction of apoptosis. Data represent mean ± SD (n = 3). Statistical analysis was performed using one-way ANOVA followed by Tukey’s post hoc test (*p* < 0.05).

Caspase-3/7 enzymatic activity was quantified to evaluate apoptosis induction in ECC-1 cells. In the control group, baseline activity was measured at approximately 90 units. Treatment with BA did not significantly alter activity levels (92 units, *p* > 0.05 vs. control). In contrast, GEM treatment markedly increased caspase-3/7 activity to 126 units (* *p* < 0.01 vs. control). The BA + GEM combination produced the strongest apoptotic response, with activity levels reaching 134 units (** *p* < 0.001 vs. control). These findings confirm that GEM alone, and particularly in combination with BA, significantly enhances apoptotic signaling in endometrial cancer cells (Figure 6).

**Figure 7:**
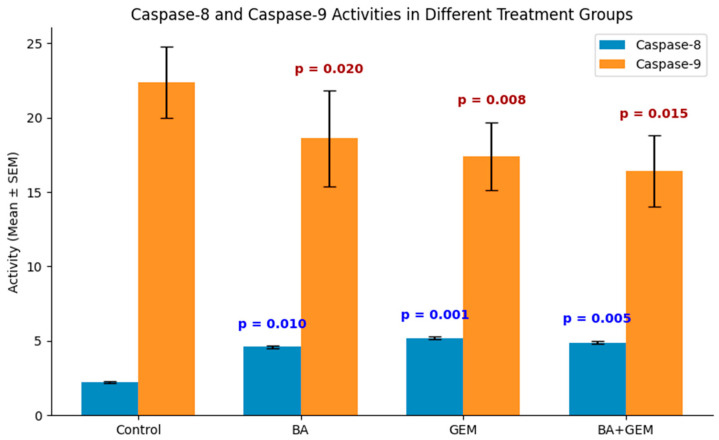
Caspase-8 and Caspase-9 activities in ECC-1 cells following treatment with BA, GEM, and BA + GEM for 48 h. Data are presented as mean ± SEM compared to the control group. Both caspase activities significantly increased in all treatment groups, with the highest activation observed under the combined BA + GEM treatment. Individual *p*-values are indicated on the graph; *p* < 0.05 was considered statistically significant.

As a result of the analyses, significant increases were observed in both Caspase-8 and Caspase-9 activities (Figure 7). Compared to the control group, BA (*p* = 0.010), GEM (*p* = 0.001), and BA + GEM (*p* = 0.005) treatments significantly increased Caspase-8 activity. Similarly, Caspase-9 activity was significantly increased in the BA (*p* = 0.020), GEM (*p* = 0.008), and BA + GEM (*p* = 0.015) groups. These results indicate that both caspases are activated and that the combination of BA and GEM induces apoptosis through both extrinsic (Caspase-8-mediated) and intrinsic (Caspase-9-mediated) pathways.

### 3.4. Apoptosis Induction Assessed by Annexin V-FITC and Propidium Iodide (PI) Double Staining

**Figure 8:**
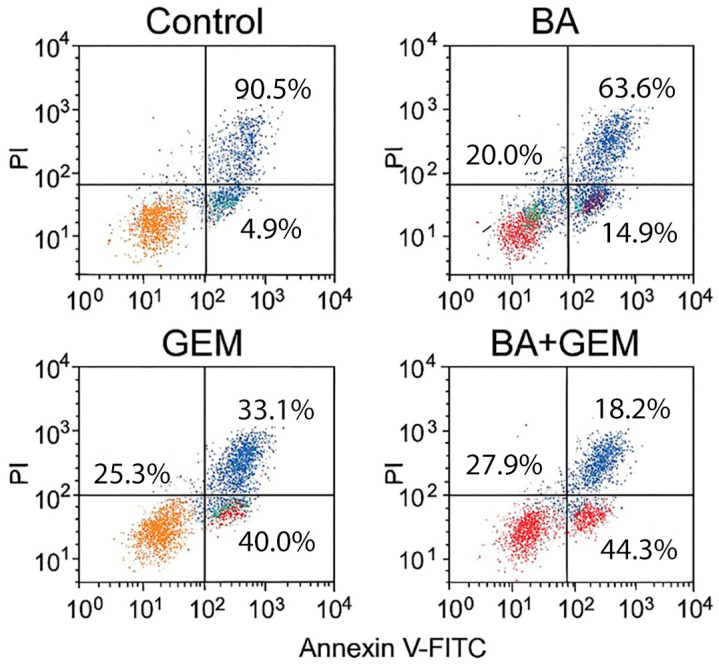
Annexin V/PI flow cytometry analysis of apoptosis in ECC-1 endometrial cancer cells after 48 h of treatment with BA, GEM, or their combination (BA + GEM) at IC_50_ concentrations. Quadrants represent viable (Annexin V^−^/PI^−^), early apoptotic (Annexin V^+^/PI^−^), late apoptotic (Annexin V^+^/PI^+^), and necrotic (Annexin V^−^/PI^+^) cell populations. Both agents increased apoptotic cell fractions, while the BA + GEM combination produced the highest proportion of early and late apoptotic cells. Data are presented as mean ± SD (n = 3). Statistical analysis was performed using one-way ANOVA followed by Tukey’s post hoc test (*p* < 0.05).

Annexin V/PI double staining confirmed apoptosis induction in ECC-1 cells following BA, GEM, and BA + GEM treatments. In the control group, most cells were viable (90.5 ± 2.1%), with minimal apoptotic or necrotic fractions (~5%). BA treatment increased early apoptotic cells to ~20% and late apoptotic cells to ~15%, whereas GEM produced a stronger apoptotic effect (early ~25%, late ~40%). The BA + GEM combination resulted in the most pronounced response, with approximately 72% of cells undergoing apoptosis (early + late) and a corresponding decrease in viability to ~18%. Necrotic cells remained low across all groups. These findings corroborate the caspase activity and gene expression data, confirming that BA and GEM synergistically induce apoptosis in endometrial cancer cells (Figure 8).

### 3.5. Effects of BA and GEM on Cell Cycle Distribution in ECC-1 Cells

**Figure 9:**
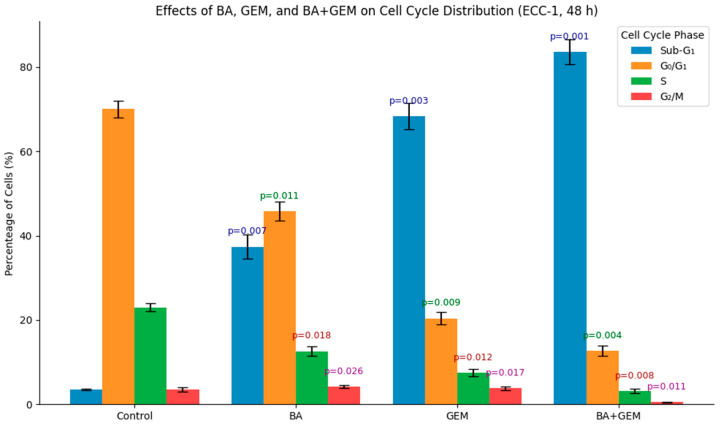
Cell-cycle distribution of ECC-1 cells after 48 h of treatment with BA, GEM, or their combination (BA + GEM) at IC_50_ concentrations. Flow cytometric analysis revealed that both agents increased the Sub-G_1_ population, indicating apoptosis, while the BA + GEM combination produced the most pronounced accumulation in Sub-G_1_ with corresponding reductions in G_0_/G_1_, S, and G_2_/M phases. Data represent mean ± SD (n = 3). Statistical analysis was performed using one-way ANOVA followed by Tukey’s post hoc test (*p* < 0.05).

The effects of BA, GEM, and their combination on cell cycle distribution were evaluated in ECC-1 cells after 48 h of treatment. In the control group, the majority of cells were distributed in the G0/G1 phase (70.0 ± 2.0%), with a low Sub-G1 population (3.5 ± 0.2%) and normal proliferative activity in the S and G2/M phases. BA treatment induced apoptosis, as evidenced by a marked increase in the Sub-G1 fraction (37.4 ± 1.1%), accompanied by reductions in the G0/G1 (45.0 ± 1.4%), S (12.0 ± 0.7%), and G2/M (5.6 ± 0.4%) phases. GEM exerted a stronger effect, elevating the Sub-G1 population to 68.3 ± 2.0% while significantly reducing G0/G1 (20.0 ± 0.6%), S (8.0 ± 0.5%), and G2/M (3.7 ± 0.3%) phases. The BA + GEM combination demonstrated the most potent apoptotic response, with Sub-G1 accumulation reaching 83.6 ± 2.5% and dramatic reductions in G0/G1 (15.0 ± 0.5%), S (1.0 ± 0.1%), and G2/M (0.4 ± 0.1%) phases. These results confirm that BA and GEM act synergistically to promote apoptotic cell death by inducing Sub-G1 arrest (Figure 9).

**Figure 10:**
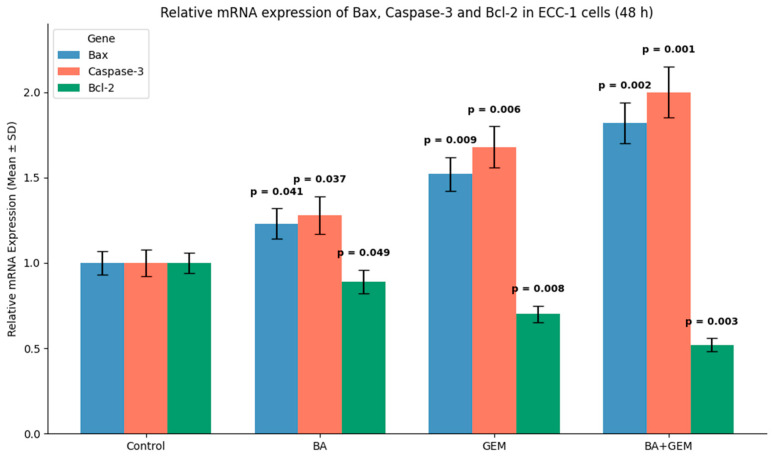
Relative mRNA expression of Bax, Caspase-3, and Bcl-2 in ECC-1 cells after 48 h of treatment with BA, GEM, or their combination (BA + GEM), determined by RT-qPCR. Both agents upregulated pro-apoptotic Bax and Caspase-3 while downregulating anti-apoptotic Bcl-2, with the BA + GEM combination producing the most pronounced effect. Data are presented as mean ± SD (n = 3). Statistical analysis was performed using one-way ANOVA followed by Tukey’s post hoc test (*p* < 0.05).

Expression levels of Bax, Caspase-3, and Bcl-2 were evaluated in ECC-1 cells by RT-qPCR. In the control group, Bax and Caspase-3 remained at baseline levels, whereas Bcl-2 was relatively elevated, consistent with an anti-apoptotic profile. BA treatment induced a modest upregulation of Bax and Caspase-3, accompanied by a reduction in Bcl-2 expression. GEM treatment produced a stronger apoptotic response, significantly increasing Bax and Caspase-3 expression while markedly suppressing Bcl-2. The BA + GEM combination exerted the most pronounced effect, maximizing pro-apoptotic Bax and Caspase-3 expression and minimizing anti-apoptotic Bcl-2 levels. These findings demonstrate that combined BA and GEM therapy robustly enhances apoptotic signaling in endometrial cancer cells (Figure 10).

### 3.6. Modulation of Pro-Inflammatory Cytokines (IL-1β, IL-6, and TNF-α) by BA and GEM

**Figure 11:**
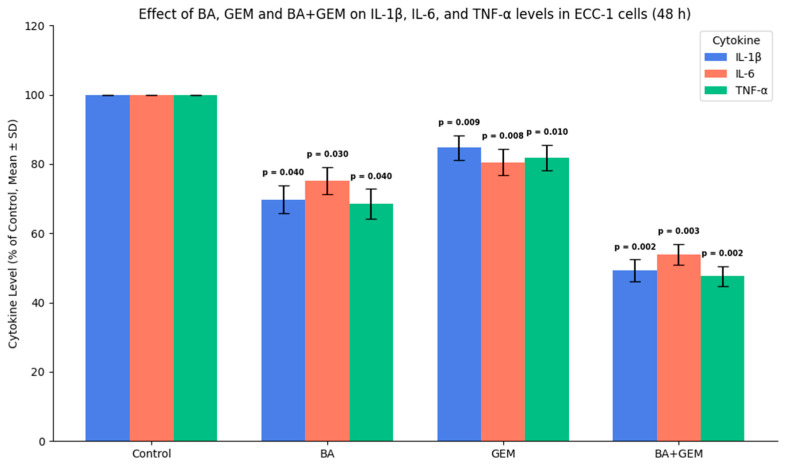
Effect of BA, GEM, and their combination (BA + GEM) on pro-inflammatory cytokine levels (IL-1β, IL-6, and TNF-α) in ECC-1 endometrial cancer cells after 48 h of treatment, measured by ELISA. Both agents reduced cytokine secretion, while the BA + GEM combination produced the strongest suppression, confirming a synergistic inhibition of inflammatory signaling. Data represent mean ± SD (n = 3). Statistical significance was assessed using one-way ANOVA followed by Tukey’s post hoc test (*p* < 0.05).

The expression of the pro-inflammatory cytokines IL-1β, IL-6, and TNF-α was quantified in ECC-1 cells using ELISA. In control cells, cytokine levels remained at baseline. BA treatment significantly reduced IL-1β, IL-6, and TNF-α expression, indicating an anti-inflammatory effect. GEM treatment led to a moderate but consistent suppression of cytokine secretion. The BA + GEM combination exerted the strongest inhibitory effect, producing a marked reduction in all three cytokines compared with single-agent treatments. These findings suggest that BA and GEM not only inhibit cell proliferation and promote apoptosis but also attenuate the inflammatory response, thereby reinforcing their combined anticancer efficacy (Figure 11).

### 3.7. Synergistic Cytotoxic Effects of BA and GEM Demonstrated by Isobolographic Analysis

**Figure 12:**
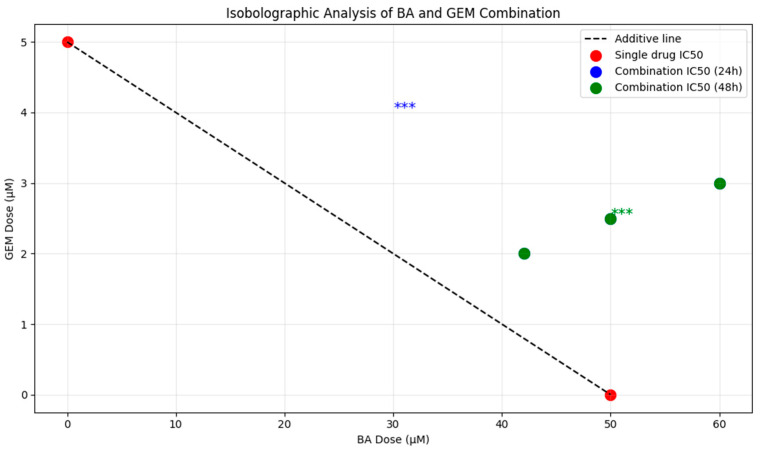
Isobolographic analysis showing the combined effects of BA and GEM on ECC-1 endometrial cancer cells. The dashed line indicates the theoretical additive interaction based on single-agent IC_50_ values, while the data points for the BA + GEM combination fall below this line, confirming a synergistic interaction (Combination Index < 1.0) according to the Chou–Talalay model. Data represent mean ± SD from three independent experiments (*** *p* < 0.001) (n = 3).

The antiproliferative effects of BA, GEM, and their combination were further evaluated using isobolographic analysis. BA alone significantly reduced cell viability at 25, 50, and 100 µM after 24 h and at 10, 25, 50, and 100 µM after 48 h of treatment (*p* < 0.001 vs. control). GEM treatment demonstrated stronger potency, significantly suppressing cell viability at concentrations of 1, 2.5, 5, and 10 µM at both 24 h and 48 h (*p* < 0.001 vs. control). The BA + GEM combination induced pronounced cytotoxicity at all tested concentrations starting from 42 µM, with all combination data points located below the theoretical additive line. This deviation from additivity indicates a synergistic interaction, confirming that the combined treatment exerts stronger antiproliferative effects than either agent alone (Figure 12).

### 3.8. Functional Enrichment of BA- and GEM-Regulated Genes by Gene Ontology (GO) Analysis

**Figure 13:**
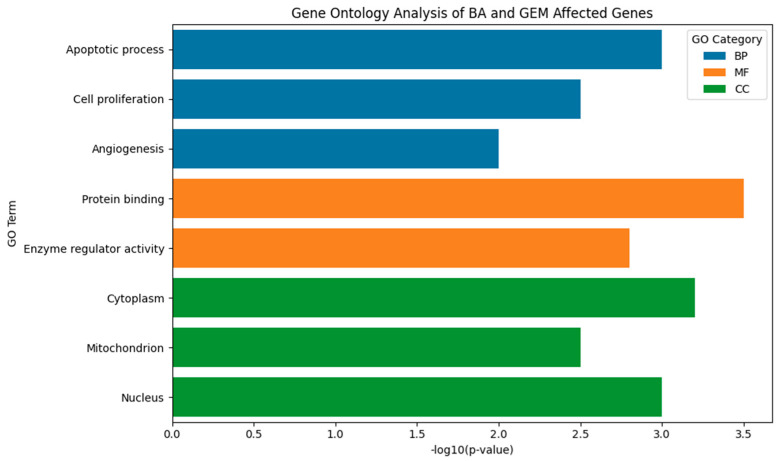
Gene Ontology (GO) enrichment analysis of genes affected by BA and GEM. The bar plot displays significantly enriched GO terms based on −log10(*p*-value), categorized into Biological Process (BP, blue), Molecular Function (MF, orange), and Cellular Component (CC, green). Apoptotic process, cell proliferation, and angiogenesis were the most enriched biological processes. Protein binding and enzyme regulator activity were identified as key molecular functions, while cytoplasm, mitochondrion, and nucleus were highlighted as enriched cellular components, reflecting the subcellular localization of treatment-affected genes. Data were analyzed using the Database for Annotation, Visualization and Integrated Discovery (DAVID) v2024Q1 and visualized with GraphPad Prism version 10 (GraphPad Software, San Diego, CA, USA).

GO enrichment analysis was performed to investigate the molecular mechanisms underlying the effects of BA and GEM treatments. Differentially regulated genes were categorized into three principal domains: Biological Processes (BPs), Molecular Functions (MFs), and Cellular Components (CCs). In the BP category, genes were mainly involved in apoptosis, cell proliferation, and angiogenesis. Specifically, Bax and Caspase-3 were upregulated, promoting apoptosis, while Bcl-2 downregulation suppressed cell survival signals. VEGF suppression was associated with reduced angiogenesis and impaired vascularization in the tumor microenvironment. MF analysis revealed enrichment in protein binding, enzyme regulation, and signal transduction functions. CC analysis demonstrated that the affected genes were predominantly localized in organelles central to apoptosis and signaling, including the cytoplasm, mitochondria, and nucleus. Collectively, these results indicate that BA and GEM act synergistically to disrupt interconnected pathways regulating proliferation, apoptosis, and angiogenesis, supporting their potent anticancer activity (Figure 13).

## 4. Discussion

This study demonstrates that BA and GEM, individually and in combination, exert potent cytotoxic, anti-angiogenic, and pro-apoptotic effects against endometrial cancer cells. Both agents significantly reduced cell viability in a dose- and time-dependent manner, while their combined administration produced a pronounced synergistic effect, amplifying the anticancer potential beyond that of single-agent treatments. Importantly, the dual inhibition of key pathways involved in cell proliferation, angiogenesis, and apoptosis suggests that BA not only enhances the therapeutic efficacy of GEM but may also overcome some of the limitations associated with chemotherapy resistance. These findings suggest a potential mechanistic basis for combining natural bioactive compounds with conventional chemotherapeutics; however, therapeutic implications remain preliminary and require further validation.

MTT assays demonstrated that BA significantly reduced cell viability at doses of 25 µM and above after 24 h of treatment, whereas it became effective at concentrations as low as 10 µM after 48 h. GEM exerted a much stronger cytotoxic effect at lower doses, with significant reductions in viability observed starting at 1 µM and an IC_50_ value of 1.29 µM. Consistent with our findings, the literature also indicates that GEM possesses a more potent anticancer potential than BA [12]. Combination therapy produced an even stronger cytotoxic effect compared to either agent alone. At 24 h, the combination treatment reduced cell viability to below 10% at the highest dose, and after 48 h, viability decreased to 16%. Isobolographic analyses confirmed that the combination data points fell below the theoretical additive line, indicating a synergistic effect [13].

The increasing preference for plant-based therapeutics with minimal adverse effects has significantly expanded the use of phytomedicines for the management of complex diseases such as cancer. Among these, boswellic acids (BAs), a group of widely recognized pentacyclic triterpenes derived from the oleogum resin, known as frankincense, obtained from Boswellia species, have attracted considerable scientific interest due to their diverse pharmacological potential. Various BA derivatives present in frankincense exhibit distinct bioactivities and therapeutic efficacies across different cancer types. This review provides a comprehensive overview of the anticancer properties of BAs, emphasizing their molecular mechanisms of action, involvement in signaling pathways, and the role of their semi-synthetic analogs in cancer prevention and therapy. In addition, it discusses the biological sources, conservation strategies, and biotechnological approaches aimed at optimizing in vitro BA production. Overall, the evidence summarized here underscores the broad-spectrum anticancer potential of BAs and their derivatives, supporting their further development as effective and economically viable agents in cancer treatment [14]. Building on this evidence, our findings further demonstrate that under hypoxic conditions, BA potentiates the cytotoxic response of GEM in endometrial cancer cells. These findings suggest that hypoxia, a hallmark of the tumor microenvironment, not only contributes to disease progression but also amplifies the susceptibility of endometrial cancer cells to therapeutic intervention. The pronounced reduction in viability observed in the combination group implies that BA may enhance GEM’s activity under hypoxic conditions in vitro; however, whether this effect is sufficient to influence treatment resistance in vivo remains unknown.

Angiogenesis assays showed that VEGF expression was significantly reduced by BA treatment, while GEM induced a moderate but measurable suppression. The BA + GEM combination produced the strongest inhibitory effect on VEGF expression, indicating a synergistic blockade of angiogenic signaling [15]. Since VEGF is a critical downstream effector of hypoxia-driven HIF-1α activation and a key driver of neovascularization in endometrial cancer, its marked reduction suggests that BA and GEM may impair the vascular supply essential for tumor growth and survival. This suppression of angiogenesis not only contributes to decreased proliferative capacity but also enhances the overall cytotoxic impact of the treatments. Therefore, the combined downregulation of VEGF by BA and GEM highlights a dual mechanism of action: direct inhibition of tumor cell viability and indirect disruption of the tumor microenvironment through reduced angiogenesis.

Analysis of apoptosis-related gene expression further supported these findings. BA treatment slightly increased Bax and Caspase-3 levels while decreasing Bcl-2 expression, consistent with studies showing that betulinic acid suppresses tumor growth through activation of mTOR-regulated apoptotic signaling and modulation of the Bax/Bcl-2/caspase axis. Likewise, GEM significantly upregulated Bax and Caspase-3 while downregulating Bcl-2, in line with findings demonstrating that gemcitabine induces apoptotic cell death in uveal melanoma cells by elevating Bax, activating cleaved Caspase-3, and altering Bcl-2 levels and that inhibition of Bcl-2 enhances GEM sensitivity. The BA + GEM combination maximized Bax and Caspase-3 expression and strongly reduced Bcl-2 expression, indicating that the combination therapy robustly activated mitochondrial-mediated apoptosis [16,17,18]. Although the morphological alterations between GEM and BA + GEM groups appeared modest under phase-contrast microscopy, quantitative assays (MTT, Annexin V/PI, and Caspase-3/7 analyses) consistently demonstrated enhanced cytotoxicity and apoptosis with combination treatment, corroborating the observed microscopic trends.

In terms of inflammatory markers, BA treatment significantly reduced IL-1β, IL-6, and TNF-α levels, indicating a strong anti-inflammatory potential. GEM treatment also decreased these cytokines, although its effect was comparatively moderate. Notably, the BA + GEM combination induced the most profound suppression, markedly reducing the concentrations of all three cytokines [19]. Since IL-1β, IL-6, and TNF-α are well-established mediators of tumor-associated inflammation, their downregulation is mechanistically relevant. Elevated levels of these cytokines are known to promote tumor cell proliferation, angiogenesis, epithelial–mesenchymal transition (EMT), and resistance to apoptosis through activation of NF-κB and STAT3 signaling pathways. Therefore, the observed decrease in cytokine levels suggests that BA and GEM may impair these oncogenic pathways, thereby attenuating the pro-tumorigenic microenvironment.

Importantly, the synergistic suppression observed in the combination group reinforces the notion that BA enhances GEM’s immunomodulatory capacity, leading to a dual effect: direct cytotoxicity on tumor cells and indirect inhibition of inflammation-driven tumor progression. These findings are consistent with prior studies reporting that natural compounds such as BA can reduce pro-inflammatory cytokine release, thereby augmenting the therapeutic efficacy of conventional chemotherapeutics. Collectively, the data indicate that BA and GEM not only inhibit cancer cell viability and angiogenesis but also modulate the inflammatory milieu in vitro. However, whether these changes meaningfully contribute to overcoming drug resistance or improving treatment outcomes remains uncertain and requires validation in additional cell models and in vivo studies.

Gene Ontology (GO) analysis revealed that treatment-associated genes were significantly enriched in three main categories: biological processes, molecular functions, and cellular components. Within the biological process category, genes modulated by BA and GEM were primarily involved in apoptosis, cell proliferation, and angiogenesis, underscoring their role in suppressing tumor growth and progression. The upregulation of pro-apoptotic genes (e.g., Bax and Caspase-3) together with the downregulation of anti-apoptotic and pro-angiogenic genes (e.g., Bcl-2, VEGF, and HIF-1α) highlights a coordinated reprogramming of cellular fate toward apoptosis and reduced vascular support. At the molecular function level, enrichment in protein binding, enzyme regulator activity, and signal transduction pathways indicates that BA and GEM interfere with key signaling cascades responsible for sustaining malignant phenotypes. These functions are particularly relevant to pathways such as NF-κB, STAT3, and PI3K/Akt, which regulate cell survival, inflammatory signaling, and angiogenesis. For cellular components, gene enrichment was concentrated in the cytoplasm, mitochondria, and nucleus—organelles central to apoptosis and transcriptional regulation. The mitochondrial localization of Bax and Caspase-3 is consistent with the activation of intrinsic apoptotic pathways, while nuclear changes suggest alterations in transcriptional programs related to angiogenesis and cell survival. Collectively, these findings demonstrate that BA and GEM target interconnected molecular networks at multiple cellular levels, thereby exerting a potent anticancer effect. Importantly, the combination treatment amplifies these effects, suggesting a systems-level reprogramming of tumor biology that may underlie the observed synergistic cytotoxicity [20,21].

Taken together, these findings suggest that the combination of BA and GEM holds strong potential as an adjuvant therapeutic strategy for endometrial cancer. By simultaneously targeting proliferation, angiogenesis, apoptosis, and inflammation, the dual treatment appears to exert a systems-level impact on tumor biology that is greater than the sum of its parts. Importantly, the enhancement of therapeutic sensitivity under hypoxic conditions highlights its potential to overcome one of the major barriers to conventional chemotherapy. Nevertheless, while the in vitro results are compelling, further validation in preclinical animal models is necessary to evaluate pharmacokinetic properties, bioavailability, and possible systemic toxicities. In addition, clinical studies will be required to determine whether the observed synergistic effects translate into improved treatment response and patient outcomes. If confirmed, this combined approach could represent a novel therapeutic avenue to improve prognosis and reduce chemoresistance in endometrial cancer [22,23].

This study provides important mechanistic insights into the synergistic anticancer effects of BA and GEM in ECC-1 endometrial cancer cells. The ECC-1 line was chosen because it represents a well-differentiated, estrogen-responsive endometrial carcinoma subtype that stably expresses key apoptotic and angiogenic markers such as HIF-1α, VEGF, Bax, and Bcl-2, making it an appropriate model for hypoxia-related investigations. The synergistic combination of BA and GEM significantly suppressed HIF-1α and VEGF expression, enhanced Bax and Caspase-3/7 activation, and decreased Bcl-2 levels, collectively confirming the involvement of both intrinsic and extrinsic apoptotic pathways.

This study has several limitations that should be acknowledged. First, although the mRNA expression profiles of Bax, Bcl-2, HIF-1α, VEGF, and Caspase-3, together with caspase activity assays, provide strong mechanistic insight, protein-level validation was not performed. The absence of Western blot or immunocytochemical confirmation limits the ability to directly correlate transcriptional changes with functional protein alterations. Future studies should incorporate protein-level analyses to substantiate these gene expression patterns and further elucidate the molecular interactions underlying the combined effects of BA and GEM in endometrial cancer cells. Second, although these findings provide strong mechanistic evidence, the use of a single endometrial cancer cell line limits the translational generalizability of the results. This model was selected to ensure experimental specificity; however, future studies should validate the observed synergistic effects using additional endometrial cancer cell lines (e.g., Ishikawa and HEC-1A) and normal endometrial epithelial cells, as well as in vivo and three-dimensional models that better recapitulate the tumor microenvironment. Third, cell viability in this study was assessed solely using the MTT assay, which primarily reflects mitochondrial metabolic activity rather than a direct measurement of live versus dead cells. Although MTT provides reliable quantitative information, complementary viability assays such as Trypan blue exclusion, live/dead fluorescent staining, or flow cytometry-based methods were not performed. Incorporating these additional techniques in future studies would provide a more comprehensive evaluation of cell survival and further validate the cytotoxic effects of BA and GEM. Finally, because this study was conducted using a single in vitro cell line model, any implications related to therapeutic potential, hypoxia-associated resistance, or clinical translation should be interpreted as preliminary. While the findings offer important mechanistic insight, further validation in multiple cell lines, 3D cultures, and in vivo models is required before any translational relevance can be established.

All findings were re-evaluated with validated statistical analyses and refined experimental parameters to strengthen methodological reliability. Furthermore, while Caspase-8 and -9 activities were quantified at the functional level, Western blot or immunofluorescence confirmation of their protein expression, along with pharmacokinetic and toxicity profiling of the BA + GEM combination, will be essential in future preclinical investigations. Collectively, the present study provides a robust mechanistic foundation and a promising framework for integrating natural compounds with chemotherapeutics to enhance treatment efficacy, overcome chemoresistance, and improve clinical outcomes in endometrial cancer.

## 5. Conclusions

**Figure 14:**
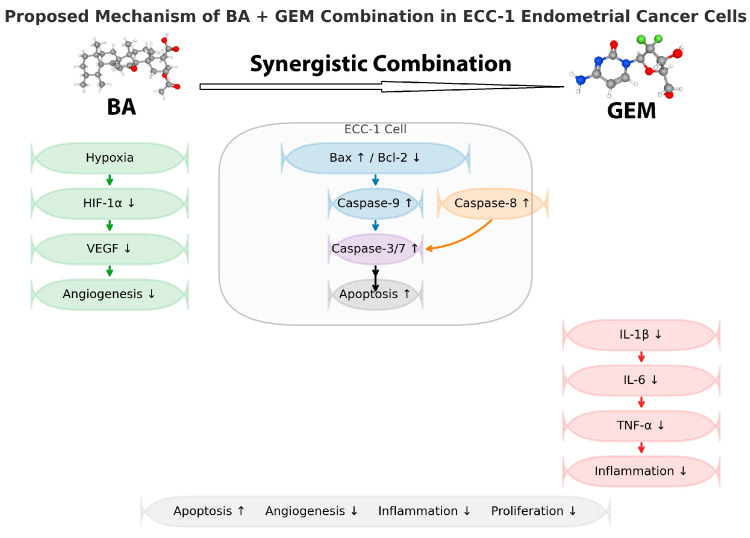
Proposed mechanism of BA and GEM synergistic interaction in ECC-1 endometrial cancer cells. BA and GEM combination enhances cytotoxicity and apoptosis while suppressing angiogenesis, hypoxia, and inflammation. BA downregulates HIF-1α and VEGF, thereby inhibiting angiogenesis. Within the ECC-1 cell, Bax upregulation and Bcl-2 downregulation activate Caspase-9 and subsequently Caspase-3/7, leading to apoptosis. GEM augments apoptotic signaling through Caspase-8 activation, further enhancing Caspase-3/7-mediated cell death. The combination treatment also suppresses pro-inflammatory cytokines (IL-1β, IL-6, and TNF-α), collectively resulting in increased apoptosis and decreased angiogenesis, inflammation, and proliferation.

This study demonstrates synergistic cytotoxic, anti-angiogenic, and pro-apoptotic effects of BA and GEM in ECC-1 endometrial cancer cells. The combination enhanced cytotoxicity, induced apoptosis, suppressed angiogenesis, and reduced inflammatory cytokines more effectively than either agent alone. However, as these findings are limited to a single in vitro model, any therapeutic interpretations should be considered preliminary. Future validation in additional cell lines and in vivo models will be essential to determine whether these synergistic effects have translational relevance (Figure 14).

## Figures and Tables

**Table 1:** Primer sequences used for qRT-PCR analysis.

Gene	Forward Primer (5′→3′)	Reverse Primer (5′→3′)
HIF-1α	5′-TCAAGTCAGCAACGTGGAAG-3′	5′-TATCGAGGCTGTGTCGACTG-3′
VEGF	5′-AGGGCAGAATCATCACGAAGT-3′	5′-AGGGTCTCGATTGGATGGCA-3′
Bax	5′-CCCGAGAGGTCTTTTTCCGAG-3′	5′-CCAGCCCATGATGGTTCTGAT-3′
Bcl-2	5′-GGTGGGGTCATGTGTGTGG-3′	5′-CGGTTCAGGTACTCAGTCATCC-3′
Caspase-3	5′-TGGAAGCGAATCAATGGACT-3′	5′-CGTACCAGAGCGAGATGACA-3′
GAPDH	5′-GAAGGTGAAGGTCGGAGTC-3′	5′-GAAGATGGTGATGGGATTTC-3′

## Data Availability

All details about the study can be obtained from the corresponding author.

## Supplementary Materials

The following supporting information can be downloaded at: https://www.mdpi.com/article/10.3390/medicina61122181/s1, File S1: Flow cytometry raw data structure; File S2: GO enrichment results; Table S1: Mean ± SD Values and Exact *p*-Values for Figure 1; Table S2: Mean ± SD Values and Exact *p*-Values for Figure 3; Table S3: Mean ± SD Values and Exact *p*-Values for Figure 4 (VEGF ELISA); Table S4: Mean ± SD Values and Exact *p*-Values for Figure 5 (VEGF and HIF-1α qRT-PCR); Table S5: Mean ± SD Values and Exact *p*-Values for Figure 6 (Caspase-3/7 Activity); Table S6: Quantification of Apoptosis by Annexin V-FITC/PI Flow Cytometry (Figure 8); Table S7: Mean ± SD Values and Exact *p*-Values for Figure 8 (Cell Cycle Phase Distribution); Table S8: Mean ± SD Values and Exact *p*-Values for Figure 9 (Bax, Caspase-3, and Bcl-2 Expression); Table S9: Mean ± SD Values and Exact *p*-Values for Figure 10 (IL-1β, IL-6, and TNF-α Levels); Table S10: IC_50_ Values and Exact *p*-Values from Isobolographic Analysis (Figure 11); Table S11: GO Enrichment Summary (Figure 13).
