# Supplementary material for: Boswellic Acid Enhances Gemcitabine’s Inhibition of Hypoxia-Driven Angiogenesis in Human Endometrial Cancer
Source: Medicina (Kaunas). 2025 Dec 8;61(12):2181. doi: 10.3390/medicina61122181 (PMC12735310; doi:10.3390/medicina61122181)
Supplement: Supplementary file 1 [file medicina-61-02181-s001.zip › Table S1 Figure1 Exact p values.pdf]

Table S1. Mean ± SD Values and Exact p-Values for Figure 1

A. Boswellic Acid (BA)

Concentration (µM)	24 h Mean ± SD (%)	48 h Mean ± SD (%)	Exact p-Value vs Control
0 (Control)	100 ± 3	100 ± 3	–
10	85 ± 4	78 ± 3	p = 0.031 / 0.018
25	70 ± 3	60 ± 3	p = 0.007 / 0.004
50	50 ± 4	35 ± 3	p = 0.0012 / 0.0009
100	30 ± 3	25 ± 2	p = 0.0004 / 0.0003

B. Gemcitabine (GEM)

Concentration (µM)	24 h Mean ± SD (%)	48 h Mean ± SD (%)	Exact p-Value vs Control
0 (Control)	100 ± 3	100 ± 3	–
1	78 ± 3	65 ± 3	p = 0.009 / 0.006
2.5	55 ± 4	40 ± 3	p = 0.0010 / 0.0007
5	35 ± 3	20 ± 2	p = 0.0004 / 0.0003
10	15 ± 2	10 ± 1	p = 0.0002 / 0.00015

C. BA + GEM Combination

Combination Dose	24 h Mean ± SD (%)	48 h Mean ± SD (%)	Exact p-Value vs Control
0 (Control)	100 ± 3	100 ± 3	–
Low (BA 10 + GEM 1)	70 ± 4	55 ± 3	p = 0.006 / 0.004
Mid (BA 25 + GEM 2.5)	50 ± 3	30 ± 3	p = 0.001 / 0.0007
High (BA 50 + GEM	25 ± 2	15 ± 2	p = 0.0004 /

5)	0.00025
