# Supplementary material for: Boswellic Acid Enhances Gemcitabine’s Inhibition of Hypoxia-Driven Angiogenesis in Human Endometrial Cancer
Source: Medicina (Kaunas). 2025 Dec 8;61(12):2181. doi: 10.3390/medicina61122181 (PMC12735310; doi:10.3390/medicina61122181)
Supplement: Supplementary file 1 [file medicina-61-02181-s001.zip › Table S10 Isobolographic IC50 with Exact p values.pdf]

Figure 12. Revised Caption and Statistical Data

Isobolographic analysis showing the combined effects of BA and GEM on ECC-1 endometrial cancer cells. The dashed line indicates the theoretical additive interaction based on single-agent IC_{50} values, while the data points for the BA + GEM combination fall below this line, confirming a synergistic interaction (Combination Index < 1.0) according to the Chou–Talalay model. Data represent mean ± SD from three independent experiments (n = 3).

Table S10. IC_{50} Values and Exact p-Values from Isobolographic Analysis (Figure 11)

Condition	BA IC_{50} (µM, Mean ± SD)	GEM IC_{50} (µM, Mean ± SD)	Combination Index (CI)	Exact p-Value
BA (24 h)	41.6 ± 1.5	–	–	–
BA (48 h)	34.7 ± 1.2	–	–	–
GEM (24 h)	–	1.29 ± 0.09	–	–
GEM (48 h)	–	1.04 ± 0.07	–	–
BA + GEM (24 h)	29.4 ± 1.3	0.84 ± 0.05	< 1.0 (synergistic)	p = 0.0012
BA + GEM (48 h)	22.9 ± 1.0	0.63 ± 0.04	< 1.0 (synergistic)	p = 0.0006
