# Supplementary material for: Boswellic Acid Enhances Gemcitabine’s Inhibition of Hypoxia-Driven Angiogenesis in Human Endometrial Cancer
Source: Medicina (Kaunas). 2025 Dec 8;61(12):2181. doi: 10.3390/medicina61122181 (PMC12735310; doi:10.3390/medicina61122181)
Supplement: Supplementary file 1 [file medicina-61-02181-s001.zip › Table S11 GO Enrichment Summary Figure 12.pdf]

## Table S11. GO Enrichment Summary (Figure 13)

Figure 13. Gene Ontology (GO) enrichment analysis of genes affected by BA and GEM. The bar plot displays significantly enriched GO terms based on −log10(p-value), categorized into Biological Process (BP, blue), Molecular Function (MF, orange), and Cellular Component (CC, green). Apoptotic process, cell proliferation, and angiogenesis were the most enriched biological processes. Protein binding and enzyme regulator activity were identified as key molecular functions, while cytoplasm, mitochondrion, and nucleus were highlighted as enriched cellular components, reflecting the subcellular localization of treatment-affected genes. Data were analyzed using the Database for Annotation, Visualization and Integrated Discovery (DAVID) v2024Q1 and visualized with GraphPad Prism version 10 (GraphPad Software, San Diego, CA, USA).

### Summary of Enriched GO Terms

| GO Term | GO Category | Enrichment Score (−log10 p-value) | Biological Interpretation |
|---|---|---|---|
| Apoptotic process | BP | 3.2 | Indicates activation of programmed cell death pathways. |
| Cell proliferation | BP | 2.7 | Reflects modulation of cell growth and division. |
| Angiogenesis | BP | 2.0 | Suggests regulation of vascular development. |
| Protein binding | MF | 3.5 | Highlights altered protein–protein interaction networks. |
| Enzyme regulator activity | MF | 2.8 | Shows modulation of enzymatic control processes. |
| Cytoplasm | CC | 3.2 | Localization of affected proteins within cytoplasmic structures. |
| Mitochondrion | CC | 2.5 | Indicates mitochondrial |

| | | | |
|---|---|---|---|
| | | | involvement in apoptosis and metabolism. |
| Nucleus | CC | 3.0 | Reflects regulation of nuclear gene expression and DNA repair. |
