# Supplementary material for: Boswellic Acid Enhances Gemcitabine’s Inhibition of Hypoxia-Driven Angiogenesis in Human Endometrial Cancer
Source: Medicina (Kaunas). 2025 Dec 8;61(12):2181. doi: 10.3390/medicina61122181 (PMC12735310; doi:10.3390/medicina61122181)
Supplement: Supplementary file 1 [file medicina-61-02181-s001.zip › Table S2 Figure 3 Exact p values.pdf]

Table S2. Mean ± SD Values and Exact p-Values for Figure 3

Condition	Normoxia Mean ± SD (%)	Hypoxia Mean ± SD (%)	Exact p-Value vs Control (Hypoxia)
Control	100 ± 4	80 ± 4	–
BA	85 ± 5	60 ± 5	p = 0.004
GEM	70 ± 5	50 ± 4	p = 0.0012
BA + GEM	55 ± 4	30 ± 3	p = 0.0003
