# Supplementary material for: Boswellic Acid Enhances Gemcitabine’s Inhibition of Hypoxia-Driven Angiogenesis in Human Endometrial Cancer
Source: Medicina (Kaunas). 2025 Dec 8;61(12):2181. doi: 10.3390/medicina61122181 (PMC12735310; doi:10.3390/medicina61122181)
Supplement: Supplementary file 1 [file medicina-61-02181-s001.zip › Table S3 Figure 4 VEGF Exact p values.pdf]

Table S3. Mean ± SD Values and Exact p-Values for Figure 4 (VEGF ELISA)

Condition	VEGF Expression (Relative Units, Mean ± SD)	Exact p-Value vs Control
Control	1.50 ± 0.15	–
BA	1.00 ± 0.10	p = 0.008
GEM	1.25 ± 0.12	p = 0.034
BA + GEM	0.70 ± 0.08	p = 0.0006
