# Supplementary material for: Boswellic Acid Enhances Gemcitabine’s Inhibition of Hypoxia-Driven Angiogenesis in Human Endometrial Cancer
Source: Medicina (Kaunas). 2025 Dec 8;61(12):2181. doi: 10.3390/medicina61122181 (PMC12735310; doi:10.3390/medicina61122181)
Supplement: Supplementary file 1 [file medicina-61-02181-s001.zip › Table S4 Figure 5 VEGF HIF1a qRT PCR Exact p values.pdf]

Table S4. Mean ± SD Values and Exact p-Values for Figure 5 (VEGF and HIF-1α qRT-PCR)

Condition	VEGF Expression (Mean ± SD)	Exact p-Value vs Control	HIF-1α Expression (Mean ± SD)	Exact p-Value vs Control
Control	1.50 ± 0.20	–	2.00 ± 0.25	–
BA	1.00 ± 0.15	p = 0.009	1.20 ± 0.18	p = 0.006
GEM	1.20 ± 0.18	p = 0.035	1.50 ± 0.20	p = 0.008
BA + GEM	0.70 ± 0.10	p = 0.0007	0.80 ± 0.12	p = 0.0004
