# Supplementary material for: Boswellic Acid Enhances Gemcitabine’s Inhibition of Hypoxia-Driven Angiogenesis in Human Endometrial Cancer
Source: Medicina (Kaunas). 2025 Dec 8;61(12):2181. doi: 10.3390/medicina61122181 (PMC12735310; doi:10.3390/medicina61122181)
Supplement: Supplementary file 1 [file medicina-61-02181-s001.zip › Table S5 Figure 6 Caspase3-7 Activity with Exact p values.pdf]

Figure 6. Revised Caption and Statistical Data

Figure 6. Effect of BA, GEM, and their combination on Caspase-3/7 activity in ECC-1 endometrial cancer cells after 48 h of treatment. Caspase-3/7 activity was measured using a luminescent assay and expressed as relative luminescence units. The BA + GEM combination markedly enhanced Caspase-3/7 activation compared with single treatments, confirming a synergistic induction of apoptosis. Data represent mean ± SD (n = 3). Statistical analysis was performed by one-way ANOVA followed by Tukey's post hoc test ($p < 0.05$).

Table S5. Mean ± SD Values and Exact p-Values for Figure 6 (Caspase-3/7 Activity)

Condition	Caspase-3/7 Activity (Mean ± SD, Relative Units)	Exact p-Value vs Control
Control	90 ± 8	–
BA	100 ± 10	p = 0.21 (ns)
GEM	125 ± 9	p = 0.036 (*p < 0.05*)
BA + GEM	135 ± 10	p = 0.008 (**p < 0.01*)
