# Supplementary material for: Boswellic Acid Enhances Gemcitabine’s Inhibition of Hypoxia-Driven Angiogenesis in Human Endometrial Cancer
Source: Medicina (Kaunas). 2025 Dec 8;61(12):2181. doi: 10.3390/medicina61122181 (PMC12735310; doi:10.3390/medicina61122181)
Supplement: Supplementary file 1 [file medicina-61-02181-s001.zip › Table S6 AnnexinV PI Quantification with Exact p values.pdf]

Table S6. Quantification of Apoptosis by Annexin V-FITC/PI Flow Cytometry (Figure 8)

Annexin V/PI flow cytometry analysis of apoptosis in ECC-1 endometrial cancer cells after 48 h of treatment with BA, GEM, or their combination (BA + GEM) at IC_{50} concentrations. Quadrants represent viable (Annexin V^{-}/PI^{-}), early apoptotic (Annexin V^{+}/PI^{-}), late apoptotic (Annexin V^{+}/PI^{+}), and necrotic (Annexin V^{-}/PI^{+}) cell populations. Both agents increased apoptotic cell frac-tions, while the BA + GEM combination produced the highest proportion of early and late apoptotic cells. Data are presented as mean ± SD (n = 3). Statistical analysis was performed using one-way ANOVA followed by Tukey's post hoc test ($p < 0.05$).

Treatment Group	Viable (%)	Early Apoptotic (%)	Late Apoptotic (%)	Necrotic (%)	Exact p-Values vs Control
Control	90.5 ± 2.1	4.9 ± 0.6	3.5 ± 0.5	1.1 ± 0.2	–
BA	63.6 ± 2.4	20.0 ± 1.3	14.9 ± 1.1	1.5 ± 0.3	Early: p = 0.018; Late: p = 0.012
GEM	33.1 ± 1.8	25.3 ± 1.0	40.0 ± 1.6	1.6 ± 0.4	Early: p = 0.009; Late: p = 0.006
BA + GEM	18.2 ± 1.2	27.9 ± 1.5	44.3 ± 1.9	1.7 ± 0.3	Early: p = 0.004; Late: p = 0.002
