# Supplementary material for: Boswellic Acid Enhances Gemcitabine’s Inhibition of Hypoxia-Driven Angiogenesis in Human Endometrial Cancer
Source: Medicina (Kaunas). 2025 Dec 8;61(12):2181. doi: 10.3390/medicina61122181 (PMC12735310; doi:10.3390/medicina61122181)
Supplement: Supplementary file 1 [file medicina-61-02181-s001.zip › Table S7 Figure 9 CellCycle with Exact p values.pdf]

Figure 9. Revised Caption and Statistical Data

Cell-cycle distribution of ECC-1 cells after 48 h of treatment with BA, GEM, or their combination (BA + GEM) at IC_{50} concentrations. Flow cytometric analysis revealed that both agents increased the Sub-G_1 population, indicating apoptosis, while the BA + GEM combination produced the most pronounced accumulation in Sub-G_1 with corresponding reductions in G_0/G_1, S, and G_2/M phases. Data represent mean ± SD (n = 3). Statistical analysis was performed using one-way ANOVA followed by Tukey's post hoc test ($p < 0.05$).

Table S7. Mean ± SD Values and Exact p-Values for Figure 8 (Cell Cycle Phase Distribution)

Treatment Group	Sub-G1 (%)	G0/G1 (%)	S (%)	G2/M (%)	Exact p-Values vs Control
Control	3.2 ± 0.4	70.3 ± 2.5	20.1 ± 1.8	6.4 ± 0.5	–
BA	37.4 ± 2.8	45.8 ± 2.3	12.6 ± 1.1	4.2 ± 0.4	Sub-G1: p = 0.007; G0/G1: p = 0.011; S: p = 0.018; G2/M: p = 0.026
GEM	68.3 ± 3.1	20.4 ± 1.5	7.5 ± 0.8	3.8 ± 0.4	Sub-G1: p = 0.003; G0/G1: p = 0.009; S: p = 0.012; G2/M: p = 0.017
BA + GEM	83.6 ± 2.9	12.7 ± 1.2	3.2 ± 0.5	0.5 ± 0.1	Sub-G1: p = 0.001; G0/G1: p = 0.004; S: p = 0.008; G2/M: p = 0.011
