# Supplementary material for: Boswellic Acid Enhances Gemcitabine’s Inhibition of Hypoxia-Driven Angiogenesis in Human Endometrial Cancer
Source: Medicina (Kaunas). 2025 Dec 8;61(12):2181. doi: 10.3390/medicina61122181 (PMC12735310; doi:10.3390/medicina61122181)
Supplement: Supplementary file 1 [file medicina-61-02181-s001.zip › Table S8 Figure 10 Bax Caspase3 Bcl2 with Exact p values.pdf]

Figure 10. Revised Caption and Statistical Data

Relative mRNA expression of Bax, Caspase-3, and Bcl-2 in ECC-1 cells after 48 h of treatment with BA, GEM, or their combination (BA + GEM), determined by RT-qPCR. Both agents upregulated pro-apoptotic Bax and Caspase-3 while downregulating anti-apoptotic Bcl-2, with the BA + GEM combination producing the most pronounced effect. Data are presented as mean ± SD (n = 3). Statistical analysis was performed using one-way ANOVA followed by Tukey's post hoc test ($p < 0.05$).

Table S8. Mean ± SD Values and Exact p-Values for Figure 9 (Bax, Caspase-3, and Bcl-2 Expression)

Treatment Group	Bax (Mean ± SD)	Caspase-3 (Mean ± SD)	Bcl-2 (Mean ± SD)	Exact p-Values vs Control
Control	1.00 ± 0.07	1.00 ± 0.08	1.00 ± 0.06	–
BA	1.23 ± 0.09	1.28 ± 0.11	0.89 ± 0.07	Bax: p = 0.041; Caspase-3: p = 0.037; Bcl-2: p = 0.049
GEM	1.52 ± 0.10	1.68 ± 0.12	0.70 ± 0.05	Bax: p = 0.009; Caspase-3: p = 0.006; Bcl-2: p = 0.008
BA + GEM	1.82 ± 0.12	2.00 ± 0.15	0.52 ± 0.04	Bax: p = 0.002; Caspase-3: p = 0.001; Bcl-2: p = 0.003
