# Supplementary material for: Boswellic Acid Enhances Gemcitabine’s Inhibition of Hypoxia-Driven Angiogenesis in Human Endometrial Cancer
Source: Medicina (Kaunas). 2025 Dec 8;61(12):2181. doi: 10.3390/medicina61122181 (PMC12735310; doi:10.3390/medicina61122181)
Supplement: Supplementary file 1 [file medicina-61-02181-s001.zip › Table S9 Figure 11 Cytokine Levels with Exact p values.pdf]

Figure 11. Revised Caption and Statistical Data

Effect of BA, GEM, and their combination (BA + GEM) on pro-inflammatory cytokine levels (IL-1β, IL-6, and TNF-α) in ECC-1 endometrial cancer cells after 48 h of treatment, measured by ELISA. Both agents reduced cytokine secretion, while the BA + GEM combination produced the strongest suppression, confirming a synergistic inhibition of inflammatory signaling. Data rep-resent mean ± SD (n = 3). Statistical significance was assessed using one-way ANOVA followed by Tukey's post hoc test ($p < 0.05$).

Table S9. Mean ± SD Values and Exact p-Values for Figure 10 (IL-1β, IL-6, and TNF-α Levels)

Treatment Group	IL-1β (Mean ± SD, %)	IL-6 (Mean ± SD, %)	TNF-α (Mean ± SD, %)	Exact p-Values vs Control
Control	100.0 ± 5.0	100.0 ± 4.8	100.0 ± 4.9	–
BA	69.8 ± 4.1	75.2 ± 3.9	68.5 ± 4.3	IL-1β: p = 0.043; IL-6: p = 0.037; TNF-α: p = 0.045
GEM	84.7 ± 3.6	80.5 ± 3.8	81.9 ± 3.7	IL-1β: p = 0.009; IL-6: p = 0.008; TNF-α: p = 0.010
BA + GEM	49.2 ± 3.2	53.8 ± 3.0	47.6 ± 2.8	IL-1β: p = 0.002; IL-6: p = 0.003; TNF-α: p = 0.002
